# Use of transcriptional age grading technique to determine the chronological age of Sri Lankan *Aedes aegypti* and *Aedes albopictus* females

**DOI:** 10.1186/s13071-021-04994-x

**Published:** 2021-09-26

**Authors:** Thilini Chathurika Weeraratne, S. H. P. Parakrama Karunaratne, Lisa Reimer, W. A. Priyanka P. de Silva, Charles S. Wondji

**Affiliations:** 1 Department of Zoology, Faculty of Science, University of Peradeniya, Peradeniya, Sri Lanka; 2 Vector Biology Department, Liverpool School of Tropical Medicine, Pembroke Place, Liverpool, L3 5QA UK; 3 Medical Entomology Department, Centre for Research in Infectious Diseases (CRID), Yaoundé, Cameroon

**Keywords:** *Aedes aegypti*, *Aedes albopictus*, Transcriptional age grading, Chronological age, Sri Lanka, Multivariate calibration modes

## Abstract

**Background:**

*Aedes aegypti* and *Ae. albopictus* are important vectors of human diseases such as dengue, chikungunya, and zika. In Sri Lanka, they have been responsible for transmitting dengue virus. One of the most important parameters influencing the likelihood of arbovirus transmission is the age structure of the mosquito population. However, mosquito age is difficult to measure with accuracy. This study aims to construct multivariate calibration models using the transcriptional abundance of three age-responsive genes: *Ae15848* (calcium-binding protein), *Ae8505* (structural component of cuticle), and *Ae4274* (fizzy cell cycle/cell division cycle 20).

**Methods:**

The transcriptional age-grading technique was applied to determine the chronological age of *Ae. aegypti* and *Ae. albopictus* female mosquito populations from Sri Lanka using the age-responsive genes *Ae15848*, *Ae8505*, and *Ae4274*. Furthermore, *Ae. aegypti* samples obtained from colonies reared at two temperatures (23 and 27 °C) were used to investigate the influence of temperature on this age-grading technique. Expression levels of these three genes were quantified using reverse transcription qualitative PCR (qRT-PCR), and results were normalized against the housekeeping gene *ribosomal gene S17* (*RpS17*).

**Results:**

The expression of *Ae15848* and *Ae8505* decreased with the age of mosquitoes and showed the most significant and consistent change while expression of *Ae4274* increased with age. The multivariate calibration models showed > 80% correlation between expression of these age-responsive genes and the age of female mosquitoes at both temperatures. At 27 °C the accuracy of age predictions using the models was 2.19 (± 1.66) days and 2.58 (± 2.06) days for *Ae. aegypti* and *Ae. albopictus* females, respectively. The accuracy of the model for *Ae. aegypti* at 23 °C was 3.42 (± 2.74) days.

**Conclusions:**

An adult rearing temperature difference of 4 °C (23–27 °C) did not significantly affect the age predictions. The calibration models created during this study could be successfully used to estimate the age of wild *Ae. aegypti* and *Ae. albopictus* mosquitoes from Sri Lanka.

**Graphical Abstract:**

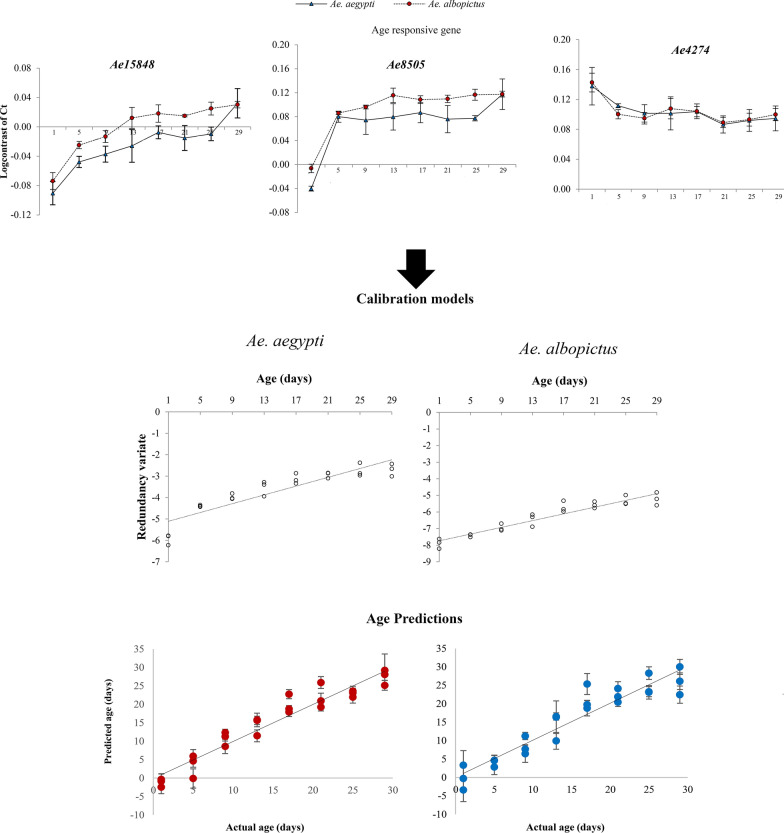

## Background

*Aedes aegypti* and *Ae. albopictus* are important vectors of human diseases such as dengue, chikungunya, and zika. Among these diseases, dengue is the most rapidly spreading mosquito-borne viral disease and is endemic in > 120 countries worldwide, including Sri Lanka. Among several factors, the age structure of the dengue vector population has been identified as one of the most important parameters influencing the epidemiology of the disease.

Based on experimental observations of the average extrinsic incubation period (EIP), the incubation period of the dengue virus within the mosquito’s body before it is transmitted to the human body is 8–12 days [1–4]. Hence, knowing the age structure of the wild populations in an area is vital in decision making, especially in vector control programmes.

As there is no specific antiviral vaccine against dengue, efficient and environmentally friendly vector control measures are needed to prevent disease outbreaks. Introduction of artificially infected *Ae. aegypti* females with *w*Mel strain of *Wolbachia* into the field has been one of the most promising dengue control interventions in the world due to failures of traditional vector control measures. This *w*Mel strain of *Wolbachia* can block the dengue virus inside the mosquito body. This environmentally friendly method of vector control has been successfully practiced in several areas in the world by introducing *Ae. aegypti* infected with *Wolbachia* (*w*Mel strain) [5]. Knowledge of the age of the vector population is important as the age affects the fitness of the released mosquitoes in the natural environment.

Morphological, biochemical, and molecular-based age-grading techniques have been developed to determine the age of female mosquitoes. However, the use of both morphological and some biochemical methods has become questionable because of their inability and inaccuracy in measuring the age of mosquitoes older than the EIP. The transcriptional age-grading technique has now been identified as the most accurate and precise approach in determining the chronological age of mosquitoes [3, 6, 7].

Quantification of expression levels of genes that show variation in their expression with the age of the female mosquito is the basis of this technique. The transcription scores of these age-responsive genes of laboratory-reared mosquitoes of known ages are then fed into a multivariate calibration model, which can later be used in age predictions of field/wild individuals. The mosquitoes used in the construction of the calibration model must be from the same mosquito strain of the field population where the researcher is planning to apply the technique [6]. The analysis of age-responsive genes during these studies have shown that this technique accurately detects the age of *An. gambiae* [8–10] and *Ae. aegypti* [2–4, 11, 12] older than 15 days, which is more than the EIP period. Trials, using mosquitoes reared in field cages, have concluded that the gene expression profiles of *Ae. aegypti* female mosquitoes could determine the age with an accuracy of ± 5 days of the actual age [2, 11, 13].

The orthologues of the eight age-responsive genes, i.e. *Ae4274*, *Ae4679*, *Ae4916*, *Ae6639*, *Ae7471*, *Ae8505*, *Ae12750*, and *Ae15848* selected from *Drosophila melanogaster*, were initially used to predict the age of female *Ae. aegypti* mosquitoes under both laboratory and field conditions [6, 14–16]. According to mosquito transcriptional age-grading studies *CG8505*/*Ae-8505*/*AAEL003259* (pupal cuticle protein 78E putative) and *SCP-1*/*Ae.-15,848*/*AAEL008844* (*calcium-binding protein, putative*) displayed the largest and most significant decrease in expression levels with the age of female mosquitoes while expression levels of fizzy/*Ae-4274*/*AAEL014025* (fizzy cell cycle/cell physiology, putative) significantly increased with age. Hence, these three genes, *Ae8505*, *Ae15848*, and *Ae4274*, have been identified as the most informative age-responsive genes that can be used in the transcriptional profiling of mosquitoes [2, 11, 13]. Cook et al. [6] have identified *Ae8505*, *Ae15848*, and *Ae4274* genes as the most reliable age-responsive genes and recommended using these three genes for future age determination studies. The gene *RpS17* (40S ribosomal protein s17), which shows insignificant variation with age, has been exclusively used as the reference gene for normalizing the samples in these studies. Furthermore, the expression of these genes is not affected by blood feeding, egg laying, digestion, and reproductive status of the mosquitoes [6, 8, 17].

However, this approach needs further validation and optimization based on the geographical region, as the age-responsive genes within these mosquito populations may have sequence polymorphisms that may affect the application of gene expression analysis. Therefore, Cook et al. [6] suggested creating separate models for mosquitoes in different geographical regions. Furthermore, fluctuations in environmental parameters, such as temperature, were flagged as being potentially important to the successful application of the technique as these may affect the transcription of age-responsive genes [6]. Hence, models constructed considering all or most of these limiting factors will increase the accuracy and precision of the transcriptional age-grading method.

At present, dengue has become one of the major causes of hospitalization and death in Sri Lanka. The largest dengue outbreak was recorded in 2017 (≈186,000 cases) in the country, which is a ≈4.3 times increase in the average number of cases that were reported from 2010 to the 2016 period [18]. Control of dengue vector populations in Sri Lanka is primarily based on the application of adulticides and larvicides. However, most of these programmes have been challenged because of the development of insecticide resistance by both dengue vectors [19, 20]. Hence, the Sri Lankan government has planned to release *w*Mel-infected dengue vectors into the field [22]. However, so far no attempts have been made in Sri Lanka to determine the chronological age and age structure of any of the dengue vectors, which is essential before implementing this programme. Both primary and secondary dengue vectors, *Ae. aegypti* and *Ae. albopictus*, are found in Sri Lanka. Multivariate calibration models constructed so far for *Ae. aegypti* have used gene transcription measures of mosquitoes collected from geographical locations where the environmental and climatic conditions are different from those of Sri Lanka. Therefore, it is essential to validate the model for the Sri Lankan mosquito population.

Although *Ae. albopictus* was considered a rural secondary dengue vector in the past, it has now invaded urban areas as well and has become the dominant *Aedes* species across most of the country [20]. There has been relatively little research into age grading of *Ae. albopictus* [21], and none is based on the transcriptional age-grading technique. Furthermore, previous studies have focused only on one species where this study focuses on two vector species belonging to the same subgenus (*Stegomyia*) from the same geographical location. This will allow us to study the suitability of transcriptional age-grading across different species within this medically important subgenus.

Hence, the current study aimed to construct multivariate calibration models using the transcriptional abundance of three age-responsive genes, *Ae15848*, *Ae8505,* and *Ae4274*, to determine the age structure of the *Ae. aegypti* and *Ae. albopictus* female mosquito population from Sri Lanka and to investigate the influence of temperature on the expression levels of these age-responsive genes of *Ae. aegypti* females.

## Methods

### Establishment of mosquito colonies and sample collection

Blood-fed mosquitoes collected from Kandy district, Sri Lanka, were used to obtain eggs to establish initial colonies of *Ae. aegypti* and *Ae. albopictus*. Colonies were maintained at an insectary with ambient conditions of 27 ± 2 °C temperature, 70 ± 10% relative humidity (RH), and a photoperiod of 12:12 (L:D); these are similar to the field site conditions.

Another *Ae. aegypti* colony (using the eggs from the above *Ae. aegypti* colony at 27 °C) was established at 23 ± 2 °C (70 ± 10% RH and 12:12 photoperiod) to determine the effect of temperature on the transcriptional age-grading technique. The larvae in all colonies were given similar conditions (similar larval densities and larval food to each tray).

Unfed adult females of both species reared at 27 °C were collected at eight time points: 1, 5, 9, 13, 17, 21, 25, and 29 days. Unfed *Ae. aegypti* females reared at 23 °C were collected at five time points: 1, 5, 9, 13, and 17 days. Thirty individuals were collected at each time point. All samples were snap-frozen at − 80 °C for molecular analysis.

### RNA extraction and cDNA synthesis

RNA was extracted from pools of ten individual mosquitoes of a given age group (3 replicates per each age class) using the Arcturus® PicoPure RNA Isolation kit (Thermo Fisher Scientific, Waltham, MA, USA) following the manufacturer's protocol. RNA was eluted in 30 µl of elution buffer provided with the isolation kit. All the steps were carried out while keeping the samples on ice, and centrifugation steps were carried out at 4 °C. After adding the wash buffer, the RNA purification columns were treated with RNase-free DNase (Qiagen Hilden Germany) and incubated for 15 min at room temperature to remove DNA contamination from the RNA samples. Samples were stored at − 80 °C for molecular analysis.

cDNA was synthesized by reverse transcription using 1 ng extracted RNA as the template. SuperScript III First-Strand Synthesis System (Invitrogen) was used for cDNA synthesis following the manufacturer's protocol. One microlitre of Oligo (dt)_20_ (50 µM), 3 µl of DEPC-treated water, and 1 µl of 10 mM dNTP mix were added to the sample and the mixture was incubated for 5 min at 65 °C. Four microlitres of 5× first-strand buffer, 1 µl of 0.1 M DDT, 1 µl of RNaseOUT, and 1.5 µl of Superscript III RT were added to the initial reaction. Sample was then incubated at 25 °C for 5 min, followed by 50 °C for 60 min and 70 °C for 15 min. The c-DNA product was treated with 1 µl Rnase H and incubated at 37 °C for 20 min to remove RNA.

### Validation and quantification of age-responsive genes using qRT-PCR assay

Three age-responsive genes, *Ae. 15848* (a gene involved in calcium-binding), *Ae. 8505* (a gene involved in producing a structural component of cuticle), *Ae. 4274* (a gene involved in fizzy cell cycle/cell physiology), and a housekeeping gene, *RpS17*, were selected for qRT-PCR analysis according to previous studies [6]. Primer sequences given in Cook et al. [6] were used to amplify these three genes in *Ae. aegypti*. Primers for *Ae. albopictus* were designed using Primer 3 software (version 0.4.0) [23, 24] (Table 1).

**Table 1:** Primer sequences and product length of the candidate genes and housekeeping genes

Gene name	*Aedes aegypti* (Primer 5’-3’)	Product length (bp)	*Aedes albopictus* (Primer 5’-3’)	Product length (bp)
*Ae 15848*	F- CGAAGAGTTCAAGGATGCCG R- TCTATGCTGACCAGACCGTC	135	F- GATGAGATCTCTGCCCTTGC R-TCGGAGTAGGACTTGCCAAC	103
*Ae 8505*	F- ATCATCTGCCAACTCCACCA R- ATCCGGCAGTCAGAGTGAAA	115	F- CGCTCAAGATAGCAACATCG R- TATGATGACGTCGCTGGTGT	130
*Ae 4274*	F- GGACGCTTAGCGGGAAGAC R- TTGGCGTTTGGGATTTACCT	81	F- GCCCGATATCATCAACGACT R- CCCTCCTTCGTTCTCGTACA	142
*RSp17*	F- GCAGCTGGACTTCAACAACA R- AACAACATCCCAACTGCACC	139	F- CAGGTCCGTGGTATCTCCAT R- TCCACTTCGATGATGTCCTG	104

*F* forward, *R* reverse

After validating the three age-responsive genes and the housekeeping gene of both species, quantitative real-time polymerase chain reaction (qRT-PCR) assays were used to quantify the expression levels of all these genes using the MX3005 qPCR system (Agilent Technologies). Each qRT-PCR reaction mixture (20 µl) was prepared using 1 µl cDNA, 10 µl Brilliant green Ultra-fast SyBr Green qPCR master mix (Agilent), 0.6 µl of primer (10 mM), and 7.8 µl nuclease-free water. The thermal cycling conditions were conducted with denaturation at 95 °C for 3 min followed by 40 cycles of 10 s at 95 °C, 10 s at 60 °C, and a last step of 95 °C for 1 min, 55 °C for 30 s, and 95 °C for 30 s. Three biological and three technical replicates were performed for each age class and each gene.

### Data analysis

Data were analyzed using MxPro qPCR software (Agilent Technologies) to obtain the Cycle Threshold (Ct) value, which is the measure of the expression of genes. Fold change (FC) of each age-responsive gene at each time point, relative to the 1-day-old mosquitoes, was calculated using the 2^−ΔΔCT^ method [25] incorporating the PCR efficiency. Gene expression value/Ct value of each candidate gene at each time point was normalized to the reference gene (*RpS17*) by calculating the log contrast values using the equation given below [6].

Log contrast *X*i = log 10 [(*X*_i_ /*X*_total_)/(*X*_ref_/*X*_total_)].

*Where, X*_i_ = mean Ct value of gene *X.*

*X*_ref_ = mean Ct of the reference gene *RpS17.*

*X*_total_ = sum of the Ct values for all genes from an individual.

One-way ANOVA was conducted (using the transcriptional abundance data/log contrast values of each experiment) to determine any significant variation in the expression of the gene with the age of mosquitoes using MiniTab 15.

A multivariate calibration method described by Cook et al. [3, 6] was used to predict mosquito age. Canonical redundancy analysis was conducted for each experimental design using the normalized gene expression values (log contrast) of all three candidate genes using the SAS statistical software (SAS University edition). Syntax previously written by Cook et al. [6] was modified and used in all the analyses. A calibration model, which explains the strength of the linear relationship among the expression of all three candidate genes and mosquito age, was constructed using the linear regression of the first redundancy variate generated during the redundancy analysis. A nonparametric bootstrapping (1000 bootstraps) method was used to assess the sampling error [95% confidence intervals (CI)] to validate the constructed calibration models and to predict mosquito pool ages. The median point of the CI intervals was considered as the likely predicted ages of mosquitoes [6]. The residual value (difference between the predicted age and actual age) was used to assess the accuracy of each model. The expression data of one experimental design were cross-checked with other models, e.g. *Ae. albopictus* age was predicted using the *Ae. aegypti* model and vice versa, to determine the possible use of one model for age prediction.

## Results

### Expression levels of age-responsive genes of *Ae. aegypti* and *Ae. albopictus* at 27 °C

**Fig. 1:**
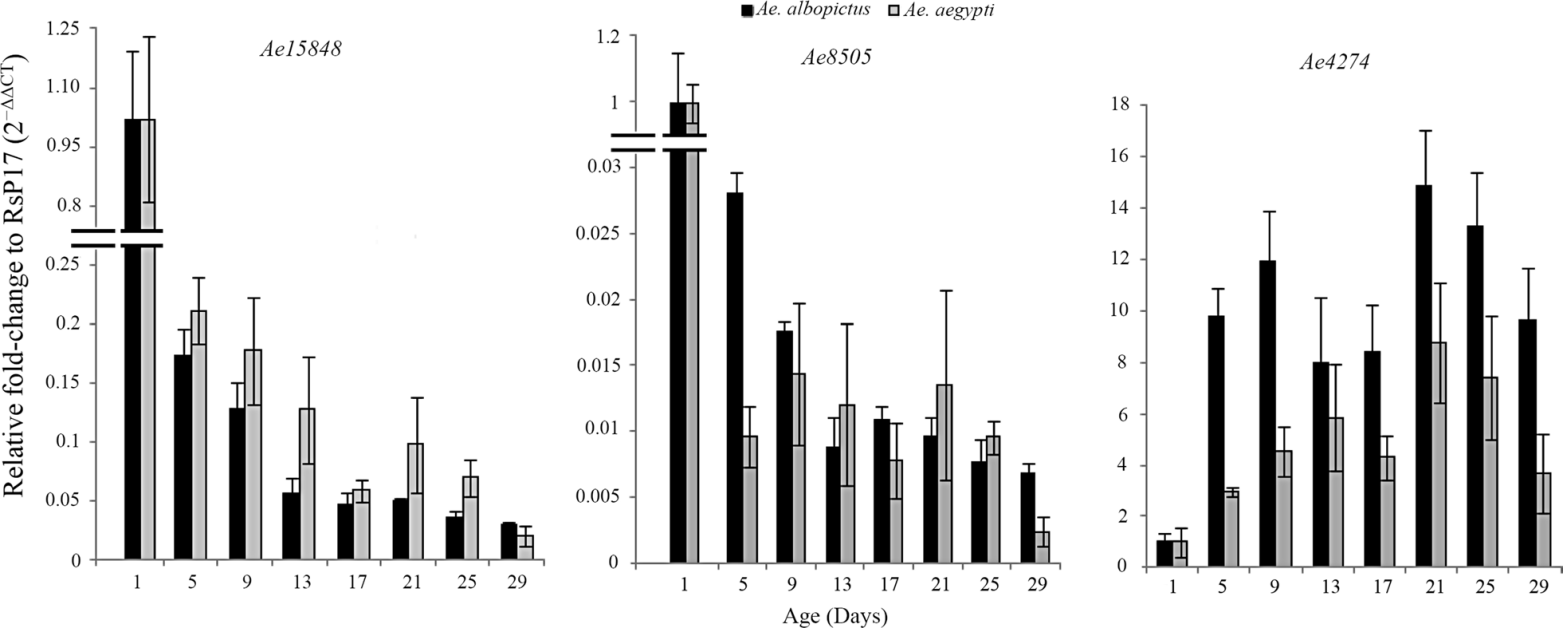
Fold change of age-responsive genes *Ae15848*, *Ae8505*, and *Ae4274* relative to the expression of 1-day-old mosquitoes of *Ae. aegypti* and *Ae. albopictus*, as determined by qRT-PCR analyses (error bars represent SEM)

All three genes, *Ae15848*, *Ae8505*, and *Ae4274*, were differentially expressed in both species and the fold changes relative to the expression of 1-day-old mosquitoes obtained are presented in Fig. 1. Underexpression of both *Ae15848* and *Ae8505* in all the age groups compared to day 1 and a strong drop in expression were observed from day 1 to day 5 for both species. The fold changes of genes *Ae15848* and *Ae8505* of 5-day-old mosquitoes compared to day 1 of *Ae. aegypti* were respectively 0.2 and 0.01. For *Ae. albopictus* day-5 FC values relative to day 1 for *Ae15848* and *Ae8505* respectively were 0.10 and 0.03. For *Ae. aegypti*, the FC of gene *Ae4274* was highest at day 21 (FC = 8.80) and the lowest FC of 2.94 at day 5. *Aedes albopictus* showed the lowest FC at day 13 (FC = 7.97) while the largest FC of 14.88 at day 21.

**Fig. 2:**
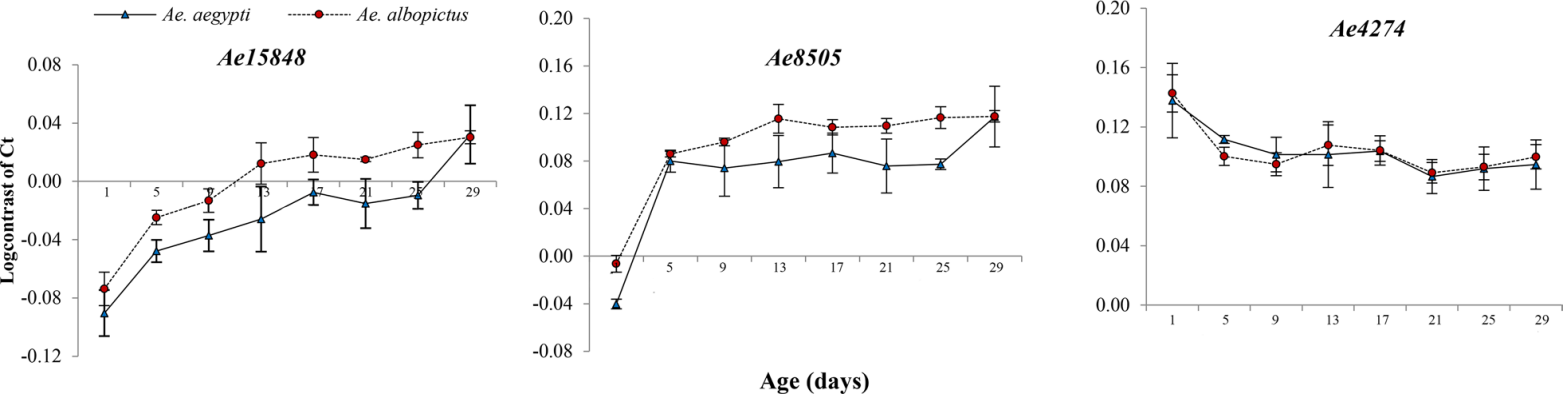
Log contrast values obtained for the three age-responsive genes *Ae15848*, *Ae8505*, and *Ae4274* at each time point of *Ae. aegypti* and *Ae. albopictus* female mosquitoes reared at 27 °C temperature

The transcriptional profiles and abundance values (log contrast of Ct) of each candidate gene at each experimental design are shown in Fig. 2. The expression levels are inversely proportional to the log contrast of normalized Ct values/transcriptional abundance. The lowest log contrast value of *Ae15848* gene for both *Ae. aegypti* [(− 0.090) ± 0.016] and *Ae. albopictus* [(− 0.074) ± 0.012] was at day 1 and highest was at day 29 (*Ae. aegypti* = 0.032 ± 0.033 and *Ae. albopictus* = 0.30 ± 0.004). The transcriptional abundance of genes *Ae15848* and *Ae8505* increased with the age of both species, which indicates the expression of both genes decreased with mosquito age. Expression levels of *Ae15848* were always greater than those of *Ae8505* at each time point.

The expression of *Ae4274* increased with the age of mosquitoes unlike the other two genes. For both mosquito species the lowest expression of *Ae4274* was at day 1 (0.138 ± 0.025 and 0.143 ± 0.013 respectively for *Ae. aegypti* and *Ae. albopictus*). The highest expression was at 21-day-old age class, and the log contrast values were 0.087 ± 0.012 for *Ae. aegypti* and 0.89 ± 0.007 for *Ae. albopictus*. The expression levels of this gene were lower compared to *Ae15848* and *Ae8505* for almost all time points tested.

According to one-way ANOVA test results, all three genes showed a significant change in expression with the age of female mosquitoes (Table 2). *Aedes aegypti* showed a significant and more consistent change in expression of genes *Ae15848* (*F* = 12.34, *p* = 0.0001) and *Ae8505* (*F* = 19.62, *p* = 0.0001) than *Ae4274* (*F* = 8.50, *p* = 0.001). *Aedes albopictus* also had a similar pattern of change in the expression of all the genes of the mosquito (*Ae1848* *F* = 43.94, *p* = 0.0001, *Ae8505* *F* = 52.04, *p* = 0.0001 and *Ae4274* *F* = 9.12, *p* = 0.001). Analysis using two-way ANOVA showed that the expression of *Ae15848* (*F* = 34.99, *p* = 0.0001) and *Ae8505* (*F* = 36.69, *p* = 0.0001) was significantly greater in *Ae. aegypti* than that for *Ae. albopictus* while *Ae4274* did not show any significant difference in its expression between these two species (*F* = 0.37, *p* = 0.547).

**Table 2:** Results of one-way ANOVA of *Ae. albopictus* and *Ae. aegypti* from different experimental designs

Gene	F value			
	*Ae. aegypti* Eight age classes	*Ae. albopictus* Eight age classes	*Ae. aegypti* in 23 °C, five age classes	*Ae. aegypti* in 27 °C five age classes
*Ae15848*	12.34*	43.94*	42.44*	14.41*
*Ae8505*	19.62*	52.04*	11.55*	30.74*
*Ae4274*	8.50*	9.12*	10.46*	5.73*

^*^Significant (*p* < 0.05)

### Expression levels of age-responsive genes of *Ae. aegypti* at 23 °C

**Fig. 3:**
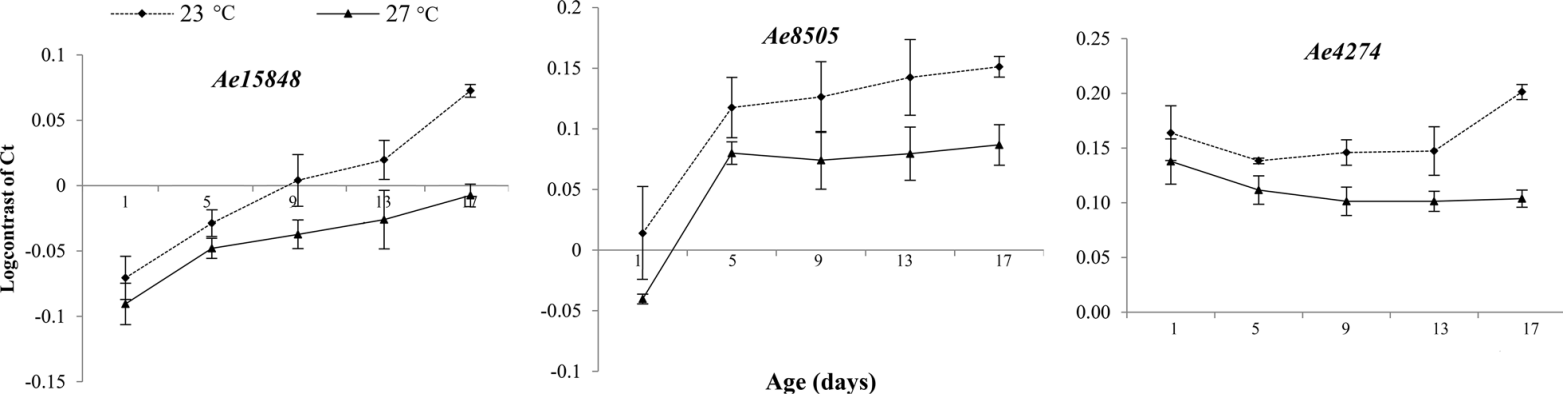
Log contrast values obtained for the three age-responsive genes, *Ae15848*, *Ae8505*, and *Ae4274*, at five age classes (1, 5, 9, 13, and 17 days) of *Ae. aegypti* females reared at 27 and 23 °C

Expression levels of the three age-responsive genes, *Ae15848*, *Ae8505*, and, *Ae4274*, were determined at 23 °C for five age classes (1, 5, 9, 13, and 17 days) of female *Ae. aegypti* and the transcriptional profiles obtained are shown in Fig. 3. The expression patterns of all three genes at 23 °C were similar to those of *Ae. aegypti* mosquitoes at 27 °C, that is, the expression of *Ae15848* and *Ae8505* decreased with mosquito age while *Ae4274* showed an increase in its expression with the mosquito age. The expression of the *Ae15848* gene was highest at all five time points (log contrast value ranging from 0.073 ± 0.005 at day 29 to − 0.071 ± 0.017 at day 1) compared to the other two genes. The transcriptional abundance of *Ae8505* was highest in 29-day-old mosquitoes (0.151 ± 0.009) and lowest in 1-day-old mosquitoes (0.014 ± 0.038). The expression of *Ae4274* increased with the mosquitoe age at both temperatures (log contrast values ranging from 0.138 ± 0.013 to 0.201 ± 0.008). Similar to 27 °C results, the change in expression levels of the three genes with female mosquito age was significant at 23 °C (Table 2). *Ae15848* (23 °C *F* = 42.44, *p* = 0.0001) showed a more consistent change in its expression with mosquitoe age compared to *Ae8505* (23 °C *F* = 11.55, *p* = 0.0001) and *Ae4274* (23 °C *F* = 10.46, *p* = 0.001). Furthermore, the transcriptional abundance was greater for *Ae. aegypti* at 23 °C compared to 27 °C indicating a lower expression at 23 °C (Fig. 3).

### Multivariate calibration models and age predictions

#### *Aedes aegypti* and *Ae. albopictus* from 27 °C

**Fig. 4:**
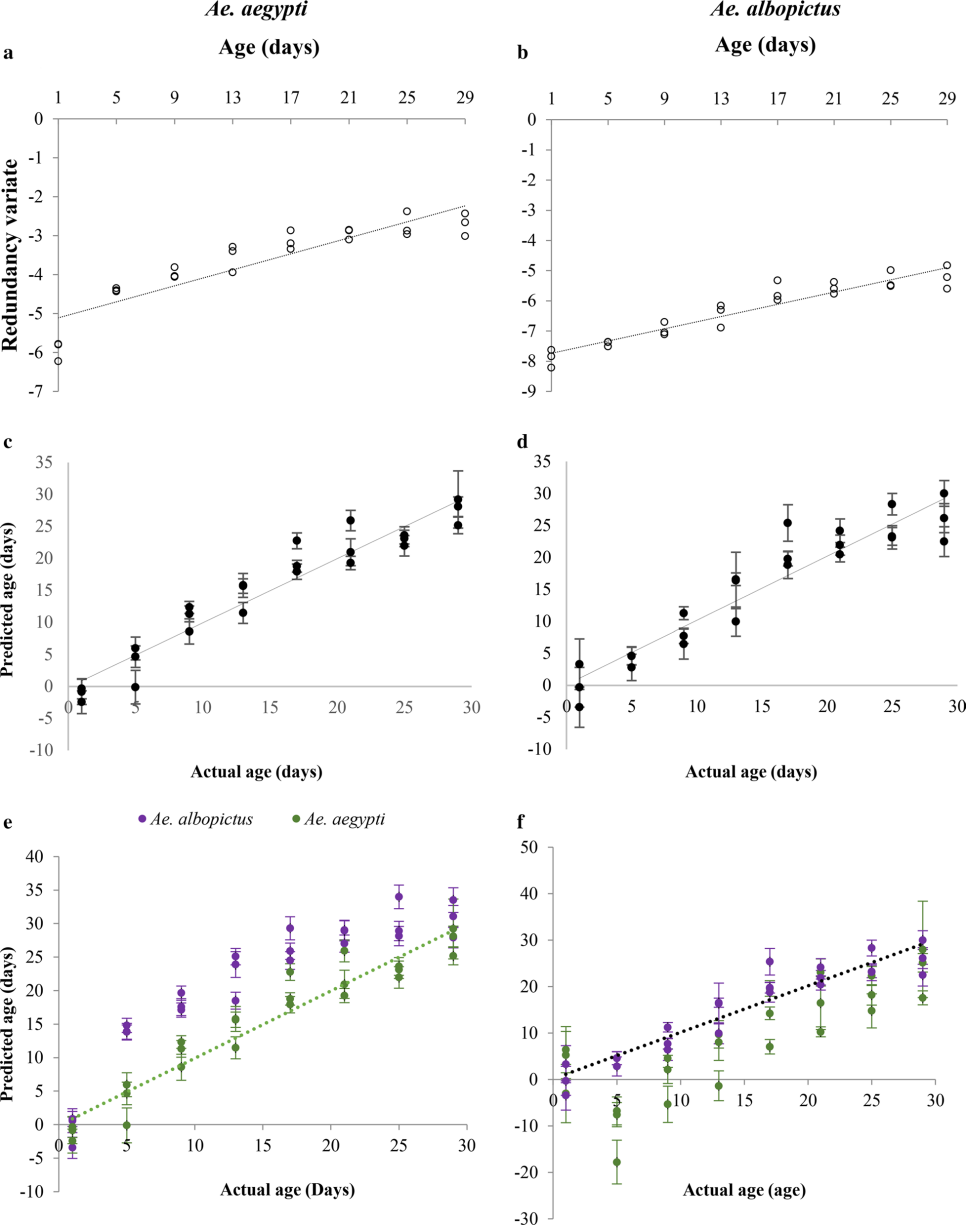
Calibration models generated for **a** *Ae. aegypti* and **b** *Ae. albopictus* female mosquitoes using the transcriptional profiles of the three age-responsive genes, *Ae15848*, *Ae8505*, and *Ae4274* c). Age predictions of **c** *Ae. Aegypti*, **d** *Ae. albopictus* female mosquitoes in eight age class; cross validation, **e** *Ae. albopictus* data checked on *Ae. aegypti* model **f** *Ae. aegypti* data checked on *Ae. albopictus* model. (Dashed lines indicate where predicted age equals actual age)

Calibration models were generated separately for each species using multivariate canonical redundancy analysis of the transcriptional abundance of the three genes, *Ae15848*, *Ae8505*, and *Ae4274*. Figure 4a and b shows the graphs drawn between the first redundancy variate and the actual age of *Ae. aegypti* and *Ae. albopictus* females.

A nonparametric bootstrapping (1000 bootstraps) procedure was used to validate the model generated for each species and to obtain the 95% confidence intervals (CI) and age predictions for the mosquito pools [6]. According to the calibration model results, the actual age of both *Ae. aegypti* (*R*^2^ = 0.8108, *p* = 0.0001) and *Ae. albopictus* (*R*^2^ = 0.8990, *p* = 0.0001) females from the tested mosquito pools showed a strong positive correlation with the first redundancy variates. The graphs between the predicted ages derived from the model and the actual age of *Ae. aegypti* and *Ae. albopictus* are shown in Fig. 4c and d. The mean residual value (difference between the actual age and predicted age of mosquitoes in the pools) was 2.19 (± 1.66) for *Ae. aegypti* and 2.58 (± 2.06) days for *Ae. albopictus*.

Furthermore, to check the species specificity of the created models, data of *Ae. albopictus* were used as the test data for the model generated for *Ae. aegypti* to predict the ages of *Ae. albopictus* at each time point and vice versa. The age of *Ae. albopictus* predicted using the *Ae. aegypti* model overestimated (mean residual value of 6.77 ± 1.41 days) (Fig. 4e) the age of *Ae. albopictus* while the *Ae. albopictus* model estimated the age of *Ae. aegypti* 7.4 ± 1.41 days (residual value) lower than the actual ages of the mosquitoes in these pools (Fig. 4f).

#### *Aedes aegypti* from 27 °C and 23 °C

**Fig. 5:**
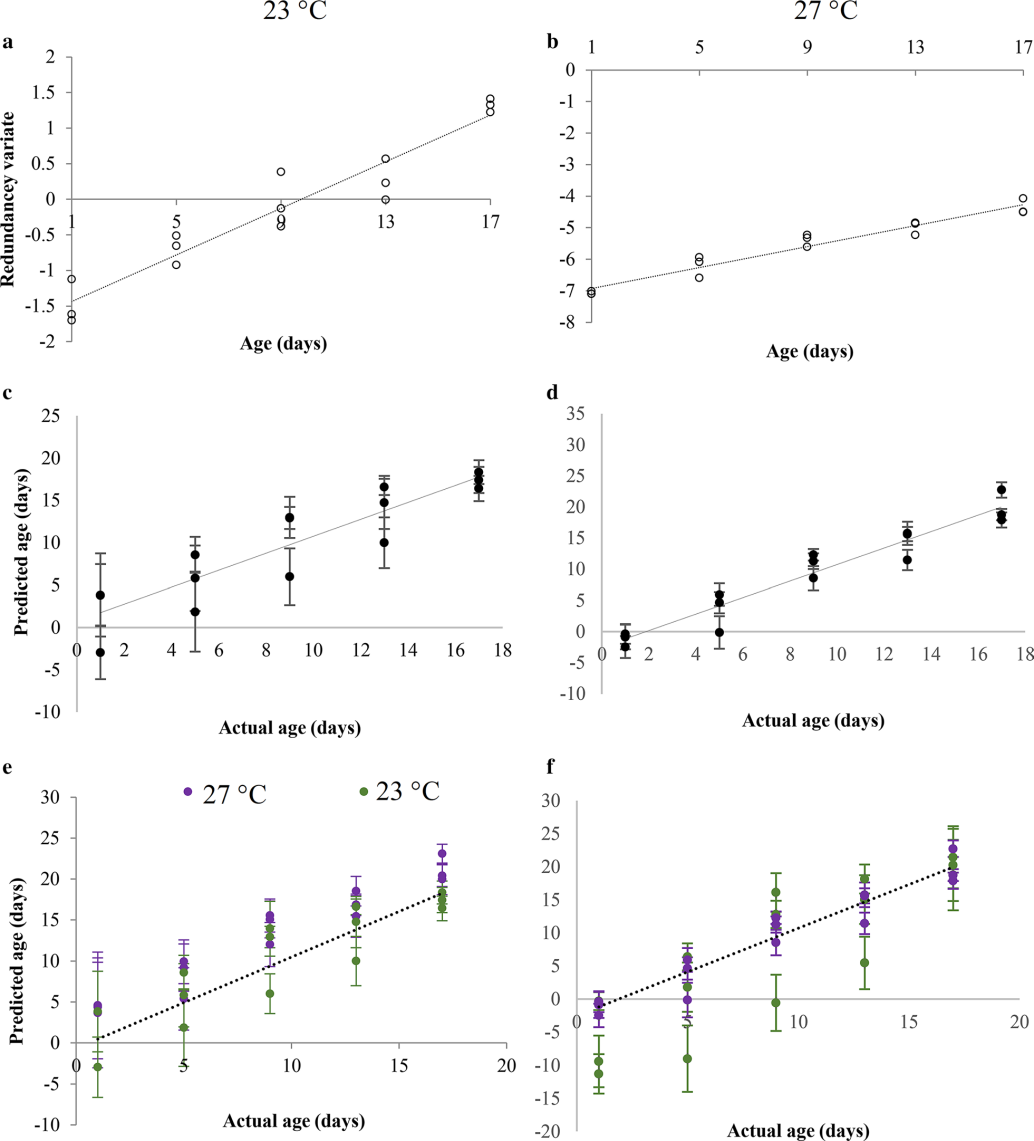
Calibration models generated for female *Ae. aegypti* mosquitoes reared at **a** 23 °C and **b** 27 °C using the transcriptional profiles of the three age-responsive genes, *Ae15848*, *Ae8505*, and *Ae4274*. **c** Age predictions of female *Ae. aegypti* mosquitoes reared at **c** 23 °C, **d** 27 °C at five age classes; cross validation, **e** 27 °C data checked on 23 °C model **f** 23 °C data checked on 27 °C model. (The dashed lines indicate where predicted age equals actual age)

Linear age prediction models were generated separately for female *Ae. aegypti* at 23 °C and 27 °C using the normalized expressions of the age-responsive genes for five age classes, 1, 5, 9, 13, and 17 days. Similar to the calibration model for mosquitoes at 27 °C, the model developed at 23 °C also showed a strong positive linear correlation with the age of female mosquitoes (23 °C; *R*^2^ = 0.9222, *p* = 0.0001) (Fig. 5a and b). According to the nonparametric bootstrap method, the residual value for *Ae. aegypti* at 27 °C was 2.19 (± 1.66) days and at 23 °C was 3.42 (± 2.74) days (Fig. 5c and d).

To test the effect of temperature on the age prediction accuracy, cross predictions were conducted. When the age of *Ae. aegypti* females reared at 27 °C were cross-predicted with the model generated for *Ae. aegypti* females at 23 °C, the age of *Ae. aegypti* was overestimated [residual value 3.93 (± 3.00) days] (Fig. 5e). The 27 °C model under-estimated (mainly the younger age classes showed the highest deviation) the age of *Ae. aegypti* females from 23 °C [residual value of 5.82 (± 3.57)] (Fig. 5f).

## Discussion

Several chemical and molecular methods have been developed to measure the age of insect vectors such as measuring pteridine concentration [26], detecting the proportions of different cuticular hydrocarbons [27], protein expression profiling [28], and near-infrared spectroscopy [28, 29]. Among these, transcriptional age grading is a molecular-based technique with high precision and accuracy that can be used to determine the chronological age of mosquitoes. However, the models developed using this approach should be validated and optimized for mosquito populations from different geographical areas and adjusted for variations of environmental factors; especially temperature prevails in such areas [6]. Hence, the present study was conducted to develop a multivariate calibration model for *Ae. aegypti* and *Ae. albopictus* from Sri Lanka using transcriptional age grading, which could later be used in estimating the age structure of wild mosquito populations. Furthermore, the study attempted to understand the effect of temperature on this age-grading technique. This study basically followed the protocol described by Cook et al. [6] with some changes in the RNA extraction and qRT-PCR technique. Also RNA was extracted from pools of mosquitoes, similar to several other studies [2, 3, 10]. Therefore, the transcriptional abundance represented by the graphs represents broad trends among a pool of mosquito.

In Sri Lanka, the highest number of yearly dengue incidences and deaths is reported from the dry zone where the annual temperature is 27.5 °C and covers a major part of the island. Furthermore, the entire country experiences relatively high humidity averaging around 80% throughout the year [30]. Hence, the mosquitoes used in the present study were maintained in insectaries with 27 ± 2 °C and 80% ± 10 relative humidity, simulating the field conditions. Transcriptional age-grading studies on *Ae. aegypti* [2, 4] and *An. gambiae* [9] have clearly shown that the models constructed for mosquitoes reared in laboratory or semi-field conditions could be successfully used to assess the age of wild mosquito populations. Therefore, the multivariate calibration models constructed during this study will be a useful tool for predicting the age of wild populations of *Ae. aegypti* and *Ae. albopictus* in Sri Lanka.

For both Sri Lankan *Ae. aegypti* and *Ae. albopictus*, the pattern of gene expression was similar to those of previous reports, i.e. reduction in the expression of *Ae15848* and *Ae8505* genes and an increase in *Ae4274* gene expression with the age of the female mosquito [3, 4, 6, 8, 11]. In all the experimental designs, the change in expression of these three genes was significantly related to the age of female mosquitoes, further proving that these are among the most informative age-responsive genes that can be successfully used in the transcriptional age-grading approach.

The expression levels of all three genes were higher in both *Ae. aegypti* and *Ae. albopictus* from Sri Lanka than in mosquito populations from other countries. However, it cannot be confirmed that these results are solely due to population differences since there are some experimental protocol differences in the present study compared to that of the previous work. Furthermore, during the current study, the expression of *Ae15848* was significantly higher than in the other two genes and showed a consistent change with mosquito age. This gene has shown a similar strong negative correlation with the chronological age of female *Ae. aegypti* [2, 4] and *An. gambiae* [9] mosquitoes from other parts of the world. *Aedes aegypti* has shown a four-fold increase in the log contrast of the same gene from 1- to 29-day-old mosquitoes in a study carried out in Northern Australia [4] and around 1.5-fold increase in mosquitoes from Central Vietnam [11] and Queensland, Australia [3]. Gene *Ae8505* of both species showed a rapid decrease in gene expression from day 1 to day 5 and more consistent change thereafter, similar to previous reports. The expression change of gene *Ae4274* with the age of mosquitoes was significant although it vaired along a small range as observed for *Ae. aegypti* females from Australia and Vietnam [3, 4, 11].

Unlike the calibration models developed previously using individual mosquitoes of *Ae. aegypti* by Cook et al. [3] (*R*^2^ = 0.73) and Hugo et al. [4] (*R*^2^ = 0.72), the two multivariate calibration models developed for both species during the present study had a strong correlation to the age of the mosquito (*R*^2^ for *Ae. aegypti* = 0.8108, *p* = 0.0001 and *R*^2^ for *Ae. albopictus* = 0.8990, *p* = 0.0001). Furthermore, in this study the accuracy of the models was greater for both species, 2.19 (± 1.66) days for *Ae. aegypti* and 2.58 (± 2.06) days for *Ae. albopictus*. These facts indicate the high precision and accuracy of the age prediction models generated during the current study. Hence, these multivariate calibration models generated from this study will be refined using data collected from mosquitoes maintained under a more natural environment. The refined models will be tested using field-collected mosquitoes. Furthermore, these findings provide strong evidence that the transcriptional age grading must be validated and optimized for mosquito strains from different geographical locations as stated by Cook et al. [6].

The age of *Ae. albopictus* was overestimated when the data were cross-predicted with the *Ae. aegypti* model and the age prediction accuracy decreases significantly from 2.58 (± 2.06) days to 6.77 (± 1.41) days. *Ae. aegypti* age will be underestimated and age prediction accuracy decreases from 2.19 (± 1.66) to 7.4 (± 3.41) days when cross-predicted with *Ae. albopictus* model. This analysis strongly supports the species-specific nature of the multivariate calibration models (although of the same genus) generated using the transcriptional age-grading technique.

Previous research work has reported a 4 °C temperature difference as the minimum range that could have a significant effect on the survival of both mosquito larvae and adults [31–34]. Hence, the model developed for *Ae. aegypti* colonies maintained at 27 °C was compared with mosquitoes reared at 23 °C (4 °C difference) to check the effect of temperature on this molecular-based age-grading approach. According to the results, the expression of the three age-responsive genes for mosquitoes from 23 °C was lower than for mosquitoes from 27 °C. Around 92% of the gene expressions were age related at 23 °C while it is 81.01% at 27 °C. According to the analysis conducted using nonparametric bootstrapping, the accuracy of age predictions did not show a considerable difference between the two temperatures (residual value 2.19 ± 1.66 days at 27 °C and 3.42 ± 2.74 days at 23 °C).

According to the results of the cross-validation, the age prediction accuracy of mosquitoes from 27 °C decreased slightly from 2.19 (± 1.66) days to 3.93 (± 3.00) days and from 3.42 (± 2.74) to 5.82 (± 3.57) days for mosquitoes reared in 23 °C. It could be stated that a temperature difference of 4 °C affects expression of age-responsive genes to a lesser extent than the difference exists between two species under the same temperature. Similar observations have been reported by Hugo et al. [4] and have suggested generating separate calibration models for inter-seasonal variations for different regions that show a greater variation in climatic factors. Sri Lanka is a small island and does not show drastic changes in temperature between areas within the country. Hence, the models developed during this study could be used to determine the age structure of wild mosquito populations from the whole country. Depending on the average temperature at the time of investigation the most appropriate model to be used can be determined.

Studies will be conducted in the future to determine the age of wild mosquitoes using the multivariate calibration models generated during this study after validating the models with mosquitoes reared under semi-field/field conditions.

## Conclusion

This is the first report from Sri Lanka on the use of the transcriptional age-grading technique to determine the age of mosquitoes and the first study to develop a model for *Aedes albopictus*. The species-specific multivariate calibration models created using the age-responsive genes, *Ae15858*, *Ae8505*, and *Ae4274*, could successfully estimate the chronological age of wild *Ae. aegypti* and *Ae. albopictus* with higher accuracy than previously reported in other mosquito species. The age of mosquitoes could be predicted with an accuracy of nearly ± 3 days of their actual age across the age spectrum. A drop in temperature from 27 to 23 °C did not have a very strong effect on the multivariate calibration models.

## Data Availability

The datasets supporting the findings of this article are included within the article.

## Abbreviations

EIP: Extrinsic incubation period
RH: Relative humidity
qRT-PCR: Quantitative real-time polymerase chain reaction
Ct: Cycle threshold
FC: Fold change
CI: Confidence interval
